# Influence of Anthropometric Height on Oculo-Manual Coordinative Reaction Time

**DOI:** 10.3390/jfmk10030334

**Published:** 2025-08-29

**Authors:** Angelo Rodio, Luigi Fattorini, Lavinia Falese, Annalisa D’Ermo, Alessandro Biffi, Fredrick Fernando, Tommaso Di Libero

**Affiliations:** 1 Department of Human Sciences, Society and Health, Sustainable Living Concept “Marco Marchetti” (Xlab), University of Cassino and Southern Lazio, Via Sant’Angelo in Theodice Campus Folcara, 03043 Cassino, Italy; a.rodio@unicas.it (A.R.); annalisa.dermo@unicas.it (A.D.); tommaso.dilibero@unicas.it (T.D.L.); 2 Dipartimento di Scienze Umane, Sociali e della Salute, European University of Technology EUt+, European Union, 10004 Troyes, France; 3 Department of Physiology and Pharmacology “Vittorio Erspamer”, Sapienza University of Rome, 00185 Rome, Italy; luigi.fattorini@uniroma.com; 4 Med-EX, Medicine & Exercise, Medical Partner Scuderia Ferrari, 00185 Rome, Italy; alessandro.biffi@med-ex.it (A.B.); fred.fernando@med-ex.it (F.F.)

**Keywords:** reaction time, visual field, coordinative abilities, dexterity, proximity sensors

## Abstract

**Objectives:** This work investigated the influence of anthropometric height on oculo-manual ability during a visuo-motor reaction time task. The aim was to determine whether aligning test configurations with individual stature changes performance outcomes. **Methods:** In the first phase, 450 participants completed a standardized reaction task using a fixed panel, and correlations were explored between anthropometric measures and performance. The results revealed significant inverse correlations between height and both reaction time total time, and reaction time intertime. A second experimental phase involved an additional group of 36 individuals, who completed the same task using both the fixed and adjustable panels, designed to align visual stimuli with each participant’s central line of sight and arm length. **Results:** A paired-sample *t*-test showed a statistically significant reduction in both reaction time total time, total time required to deactivate all 54 lights targets, (32.1±3.26 s to 30.7±2.58 s, p<0.05) and reaction time intertime, average time interval between successive light deactivations out of a total of 54 lights, (0.31±0.123 s to 0.21±0.149 s, p<0.01), time total time, total time required to deactivate all 54 lights targets, (32.1±3.26 s to 30.7±2.58 s, p<0.05) and reaction time intertime, average time interval between successive light deactivations out of a total of 54 lights, (0.31±0.123 s to 0.21±0.149 s, p<0.01) under the adjustable panel configuration. **Conclusions:** These findings suggest that standard testing configurations may disadvantage individuals with shorter stature and highlight the benefits of personalized setups for assessing and enhancing oculo-manual coordination.

## 1. Introduction

Coordinative abilities are key indicators of overall wellness and are closely linked to the functioning of the central nervous system [1], particularly through the integration of complex somatosensory information, such as inputs from the vestibular system and proprioceptive cues mediated by muscle spindles, Golgi tendon organs, and other mechanoreceptors [2,3]. Oculo-manual coordination is particularly important in the planning and execution of motor tasks, as it affects how efficiently an individual responds to visual stimuli and interacts with the surrounding environment [4]. In preventive care, rehabilitation, motor science, and occupational risk assessment, coordinative abilities represent reliable indicators of general fitness and well-being, as they are closely linked to injury risk reduction and overall quality of life [5,6]. To our knowledge, no standardized reference tables currently exist for oculo-manual coordinative performance. The only available benchmarks are derived from regression-based estimations, such as those used in the Ruler Drop Test [7], or are absent, as in the case of the Finger Tapping Test [8]. These are two commonly used tests for coordinative assessment, although they fail to account for individual functional capacity, which naturally declines with age and differs by gender [9]. Similarly, widely used hand–eye coordination tests only provide normative values stratified by age and gender, without taking into account other important anthropometric characteristics such as height, weight and derived parameters. Moreover, these tests primarily involve isolated body segments, such as the hand or fingers, and do not reflect the integration of the entire somatosensory system during dynamic motor execution. In our view, there are currently no oculo-manual coordination tests capable of involving and stressing the entire body. Since somatosensory information must interact with vestibular and proprioceptive inputs to produce an effective motor response, it is crucial to adopt test setups that involve full-body engagement, especially in an upright posture, and for motor task dynamic complexity. Another relevant aspect to consider is the visual field, which plays a fundamental role in eye-hand coordination, which refers to the spatial area perceivable by the eyes when focused [10,11]. This field is influenced by non-uniform distribution of photoreceptors on the retina [12,13], as well as by the asymmetry between the upper and lower visual fields, with the lower field generally offering greater sensitivity and spatial resolution due to its higher functional relevance in motor tasks [14]. Notably, the human retina exhibits greater sensitivity and visual acuity in the lower visual field [15,16]. For example, regarding coordinative abilities, several test modes and instruments are available that require the subject to stand upright and maintain an upright positioning of the instrument [17,18]. This fixed positioning may induce compensatory movements to reach targets depending on the subject’s height, leading to slower response times, increased neural processing demands, and reduced accuracy. Anthropometric factors such as stature, limb length, and posture relative to the visual field can therefore influence response efficiency [19,20,21]. This anatomical and functional asymmetry suggests that taller individuals may be more naturally aligned with zones of heightened visual sensitivity, particularly in standardized tests utilizing fixed panels where stimuli are presented predominantly in the lower visual quadrants [22,23,24]. Under normal conditions, the visual field extends approximately 60° nasally, 50° superiorly, 90° temporally, and about 70° inferiorly. The lower visual field, in particular, plays a crucial role in motor activities, as it contains a higher density of photoreceptors and is more sensitive to stimuli, thereby facilitating faster and more accurate motor responses [25,26]. The standardization of visual responsiveness tests often neglects anthropometric diversity, potentially disadvantaging individuals of shorter stature due to misalignment between the stimulus area and the central visual field [27]. Therefore, tailoring the target position could be useful to better account for individual characteristics. Recently, there has been growing interest in using innovative tools for evaluating coordinative abilities, such as the Batak system (Quotronics Limited, Hardware device, version Batak, United Kingdom) composed of fixed tubular frames with lights placed in predefined positions and other panels where lights are positioned arbitrarily [28,29], without following a scheme adapted to the subject’s anthropometric characteristics. Among these, tests involving vertical panels equipped with lights are increasingly used [30]. These fixed vertical structures require participants to turn off the lights as quickly as possible, allowing for the assessment of total response time and the average reaction time between each light [31]. A fixed configuration is generally adequate in assessment contexts aimed at measuring performance changes before and after an intervention [32]. However, while it is possible to characterize individual fitness levels for conditional abilities through standardized evaluations, this is not yet the case for coordinative abilities, where tests are typically used only to monitor the effects of an intervention. Therefore, current fixed-panel approaches may not accurately characterize an individual’s fitness level in terms of coordinative abilities. In such cases, it becomes crucial to adapt the positioning of visual stimuli to each subject’s anthropometric characteristics, particularly their height. Unlike more static assessment methods, which involve specific motor districts, such as the Ruler Test [33] or the Tapping Test [34], our approach engages the entire body, providing a more comprehensive evaluation of functional motor responsiveness. In light of these considerations, the present study aims to compare eye-hand coordinative performance using a fixed versus an adjustable panel in an oculo-manual coordinative abilities assessment. Specifically, we hypothesize that height significantly influences performance, and that aligning the panel height with each participant’s central visual field will enhance performance outcomes, particularly for individuals of shorter stature.

## 2. Materials and Methods

To verify our initial hypothesis regarding the influence of anthropometric parameters on oculo-manual performance, the experimental protocol was divided into two phases. The first phase involved a fixed panel (FP) configuration, described in Section 2.2, to collect baseline data and identify potential correlations between coordinative parameters and stature when using a fixed configuration. The second phase introduced an adjustable panel (AP), described in Section 2.4, to explore whether adapting the stimulus height to individual visual fields could enhance performance.

### 2.1. Participants—First Phase

The sample, comprising 450 healthy participants, included a mixed population of students and staff from the University of Cassino and Southern Lazio. Participants had no known neurological or musculoskeletal conditions that could interfere with motor performance. Anthropometric characteristics are summarized in Table 1. The distribution of the sample parameters was found to be statistically significant according to the Shapiro-Wilk normality test.

To minimize potential confounding variables, participants were asked to abstain from caffeine consumption and from engaging in intense physical activity for at least 24 h before testing. Impairment related to visual acuity and visual field was verified through self-report and color blindness using the Ishihara table. All participants were informed about how the protocol would be carried out and provided their consent before participating in the study. Moreover, informed consent and authorization about benefits and risks was obtained in accordance with the Declaration of Helsinki for Human Research of 1964. The study was approved by the Institutional Review Board of the University of Cassino and Southern Lazio (Approval No. 24777.2022.12.12).

### 2.2. Experimental Protocol—First Phase

In the first phase, participants completed a visuo-motor reaction time task using a fixed panel consisting of 12 lights targets positioned at a standard height, as described in previous work [35]. This configuration, referred to as the standard test, served as a baseline setup to assess general reaction performance and identify any limitations arising from a fixed configuration of visual stimuli, particularly concerning participants’ posture and height. Each participant completed three trials, and rest intervals were taken to avoid learning or fatigue effects (5 min); the best result was recorded.

### 2.3. Participants—Second Phase

In this second phase, an additional group of participants was enrolled through a University internship program. The sample was composed of 36 students, and all participants were male within the same age range, with good fitness levels, and in normal weight value [36]. Sample characteristics are presented in Table 2. In addition, visual acuity and visual field impairments were verified through self-reporting, and color blindness was assessed using the Ishihara table [37]. This phase of the protocol was conducted six months after the first, a period during which new participants were recruited and the adjustable panel was designed and constructed.

**Figure 1:**
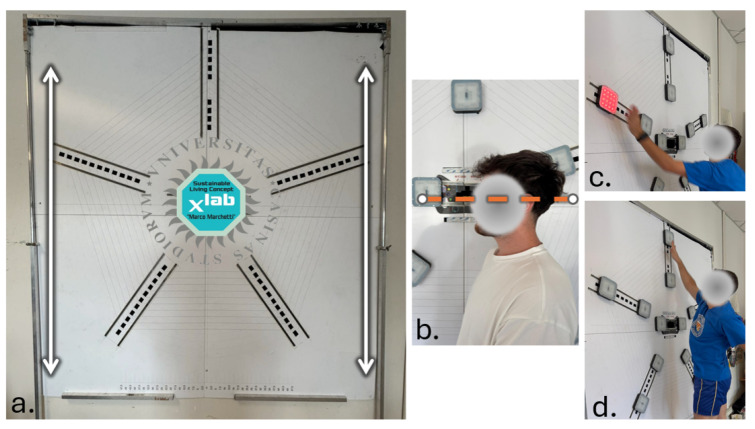
(**a**) Front view of the adjustable sliding panel. Arrows show upward/downward adjustability. (**b**) Side view showing subject’s visual alignment for panel setting. (**c**,**d**) Examples of light target layouts used in testing.

A custom-built AP configuration was developed and is now described in greater detail (Figure 1). The device consisted of a vertical Forex panel featuring ten pentagonal visual targets, arranged in a circular pattern around a central stylized element resembling a steering wheel. Each target was equipped with a multicolor LED-based proximity sensor and Bluetooth connectivity. The panel was mounted on a height-adjustable support, enabling precise vertical alignment with each participant’s eye level. This allowed for optimal positioning of the stimuli within the central and lower visual fields areas known for higher visual acuity and faster visuomotor processing thereby reducing variability due to anthropometric differences. Participants stood in front of the panel and were instructed to deactivate a sequence of 54 randomly illuminated targets as quickly and accurately as possible. After an initial start signal, a single light was activated. The participant had to promptly touch the illuminated target to deactivate it, which immediately triggered the next light to appear at a new random location. This process continued until all 54 targets had been deactivated, completing the task.

- FP: The panel and the lights were positioned to replicate the exact testing conditions used during the first phase assessment. An exception was made for the panel structure, which was designed to be horizontally translatable to accommodate the two testing configurations.

- AP: The panel was individually adjusted to align with each participant’s natural visual focus, defined as the horizontal line of sight when looking straight ahead with the head in a neutral position (166 cm ± 0.07). Anatomically, this corresponds to the primary visual axis, which extends from the center of the fovea in the retina to the point of fixation in the environment, and is considered the most accurate and sensitive area for processing visual stimuli [24,38].

Each participant completed three trials for each test configuration, with 5-min rest intervals between trials to avoid learning or fatigue effects. To further minimize potential learning effects, the order of execution with FP and AP was randomized and conducted on separate days.

### 2.4. Experimental Protocol—Second Phase

As in the first phase, the following variables were recorded, and the best-performing trial was selected for statistical analysis:

- Reaction Test Total Time $\left(RT_{TT}\right)$: total time required to deactivate all 54 lights targets.
- Reaction Test Intertime $\left(RT_{Int}\right)$: average time interval between successive light deactivations out of a total of 54 lights.

## 3. Statistical Analysis

All statistical analyses were conducted using the *Jamovi* software ((Jamovi, Version 2.4.5; The jamovi project, Sydney, Australia, https://www.jamovi.org) [39]. The Shapiro–Wilk test was used to assess the normality of the data distributions. For the first phase, as none of the main variables showed a normal distribution (*p* < 0.001), non-parametric tests were applied. Spearman’s rank correlation coefficients (ρ) were computed to examine associations between anthropometric variables (height, weight, and BMI) and coordination performance metrics, specifically RT_*TT*_ and RT_*Int*_. Linear regression analyses were performed to derive standardized regression coefficients (β) and coefficients of determination ($R^{2}$) for each anthropometric predictor. In the second phase, a statistical power analysis was conducted to determine whether the sample size was adequate (Power to detect > 80%. As the data met the assumption of normality, paired-sample *t*-tests were used to compare performance outcomes between the FP and AP conditions. Statistical significance was set at p<0.05 for all tests. Descriptive statistics are reported as mean ± SD.

## 4. Results

The Shapiro–Wilk test was used to assess the normality of the main variables. The results showed that none of the measured parameters followed a normal distribution (*p* < 0.001), thereby justifying the application of non-parametric statistical analyses in the initial phase. In the FP configuration, Spearman’s rank correlation revealed a significant and inversely proportional association between height and $RT_{TT}$ (ρ=−0.482, p<0.001), as well as between height and $RT_{Int}$ (ρ=−0.475, p<0.001), as shown in Table 3. Linear regression analyses were conducted using height, weight, and BMI as independent variables, and $RT_{TT}$ and $RT_{Int}$ as dependent variables. The linear regression analysis for $RT_{TT}$ yielded a slope coefficient of β=−0.264, while for $RT_{Int}$ the coefficient was β=−0.00547. Before interpreting the regression models, the residuals were tested for normality using the Shapiro–Wilk test. The residuals did not show significant deviations from normality (*p* > 0.05), confirming the validity of the regression assumptions. The coefficients of determination were $R^{2}=0.232$ for $RT_{TT}$ and $R^{2}=0.226$ for $RT_{Int}$, as reported in Table 3. For weight (Table 4), the correlation with $RT_{TT}$ in the overall sample was ρ=−0.222, p<0.001, with a regression coefficient of β=−0.24. Similarly, for $RT_{Int}$, the correlation with weight was ρ=−0.217, p<0.001, with β=−0.22. The corresponding $R^{2}$ values were 0.049 for $RT_{TT}$ and 0.047 for $RT_{Int}$. BMI showed a comparable pattern, although the correlation with $RT_{TT}$ was not statistically significant (p=0.219). No significant correlation was found with $RT_{Int}$ either, as shown in Table 5. The standardized regression coefficients for BMI were β=0.08 for $RT_{TT}$ and β=0.09 for $RT_{Int}$, with $R^{2}=0.003$ for both models.

In Table 6, the mean, relative standard deviation, and Shapiro-Wilk normality test results are reported for the $RT_{TT}$ and $RT_{Int}$ parameters of the reaction test. The descriptive analysis showed a normal distribution of the main variables, and consequently, a paired-sample *t*-test was conducted to compare performance between the FP and AP. The analysis revealed a statistically significant reduction in both $RT_{TT}$ and $RT_{Int}$ under the AP configuration. Specifically, $RT_{TT}$ decreased from 32.1±3.26 s (FP) to 30.7±2.58 s (AP), p<0.05, and $RT_{Int}$ decreased from 0.31±0.123 s to 0.21±0.149 s, p<0.01.

## 5. Discussion

This study aimed to investigate whether the hypothesis that eye-hand coordination parameters differ when using a FP versus an AP in oculo-manual coordinative testing. Based on the anatomical and functional asymmetries of anthropometric characteristics and of the visual field, particularly the increased receptor density in the lower visual field compared to the upper field [24,38], it was hypothesized that taller individuals would exhibit enhanced performance in tasks involving fixed-height visual stimuli in reaction time test results. $RT_{TT}$ and $RT_{Int}$ results obtained in the first phase support this hypothesis, revealing a significant inverse correlation between the anthropometric variable and oculo-manual coordinative abilities in the FP configuration. Spearman’s correlation indicated a moderately strong negative association between height with both $RT_{TT}$ and $RT_{Int}$, suggesting that taller participants completed the task more quickly and responded more promptly to stimuli. Among these, height was the parameter that most influenced performance in reaction time tests [40]. These trends were further supported by linear regression analyses, which confirmed that greater height was associated with reduced task durations ($RT_{TT}$) and shorter response latencies ($RT_{Int}$). Moreover, the weight parameter was negatively correlated with both $RT_{TT}$ and $RT_{Int}$. Although the vertical adjustment between the FP and AP configurations was relatively small (approximately 1.5 cm on average), its impact on oculo-manual performance was statistically significant. Even minor vertical displacements can shift the visual stimulus out of the lower visual field known for higher spatial resolution and faster visuomotor integration [25,38], thus affecting response times. Our results regarding anthropometric influences are consistent with prior research. Height has been associated with faster visual and auditory reaction times [19,20], while BMI often shows no clear relationship with fine motor responsiveness [21]. In our sample, BMI was not significantly correlated with RT outcomes, reinforcing its limited predictive value in oculo-manual tasks. In the second phase of the study, having found that stature significantly influenced specific coordination ability (RT parameters), a paired-sample *t*-test was conducted to compare performance between the FP and AP configurations. The results of this paired-sample *t*-test demonstrated a statistically significant improvement in both $RT_{TT}$ and $RT_{Int}$ when participants used the AP configuration. The vertical adaptive alignment of the panel’s central area was adjusted to match each participant’s visual field focus. Therefore, with the proposed configuration, the results indicated more efficient responses: the $RT_{TT}$, representing the time to turn off 54 lights, was reduced by approximately 15%, while the $RT_{Int}$, the intertime between the deactivation of one light and the next, decreased by about 6%. These findings suggest that performance in the FP configuration was negatively affected by vertical misalignment. Overall, these results support the conclusion that oculo-manual coordinative abilities may be sensitive to anthropometric variability, particularly to the vertical positioning of visual stimuli relative to the participant’s visual field. This aspect does not represent a limitation when the FP-type panel is used merely to evaluate the effects of a training program or intervention, where anthropometric variability becomes less relevant [41]. In such contexts, the primary interest lies in monitoring intra-individual progress over time rather than comparing absolute performance across different individuals. However, in contexts where the aim is to characterize individual performance levels in a specific coordinative ability, as is typically done using normative comparison tables [42], anthropometric characteristics acquire inter-individual functional significance, just as occurs with conditional abilities, and must therefore be accounted for in the evaluation process. Our modular assessment methodology, by standardizing the protocol with respect to each participant’s anthropometric features and visual field alignment, allows for a more accurate functional characterization of oculo-manual coordinative abilities. However, the limited diversity of the sample represents a limitation. Future studies will aim to include a larger and more heterogeneous sample in terms of age, gender, fitness level, and type of practiced sport. Moreover, this approach should be extended to other coordination tests in order to confirm our findings. This approach not only enables pre- and post-intervention comparisons but also supports functional assessments across diverse populations, such as sedentary individuals, the elderly, youth, and athletes. It is particularly useful for identifying differences across various sports categories, such as skill-based, endurance, power, dynamic, or static disciplines. Given that factors such as age-related decline in nerve conduction, proprioception, and muscle mass (sarcopenia), as well as sex-related differences in limb proportions and strength, can influence performance, relying solely on raw scores may be misleading [43]. In this context, oculo-manual coordinative testing offers a valuable and sensitive tool for assessing functional motor abilities. An adaptive panel configuration thus becomes essential for producing reliable normative reference values and ensuring meaningful comparisons across individuals, as it is for evaluating conditional abilities.

## 6. Conclusions

This study highlights the importance of accounting for anthropometric variability, particularly height, in the design of visuo-motor coordinative assessments. The use of a height-adjustable testing system proved effective in improving performance outcomes, supporting the need for more personalized and equitable evaluation protocols. Despite the encouraging results, some limitations must be acknowledged. In particular, the second phase of the study included only male participants, as the number of female volunteers was too low to allow for valid statistical analysis. Further studies involving more diverse populations and longitudinal designs are also recommended to evaluate the broader applicability and long-term benefits of adjustable testing systems. Additionally, the development and standardization of calibration procedures will be crucial to ensure reproducibility and reliability across various test environments. Looking forward, a key goal will be the creation of normative reference tables for coordinative ability assessments that take into account individual anthropometric characteristics, as exemplified by the approach used in our study. This step is crucial for enabling a meaningful interpretation of an individual’s motor performance.

## Figures and Tables

**Table 1:** First phase participants’ anagraphic and anthropometric characteristics.

Descriptives			Shapiro–Wilk
	**Mean**	**SD**	***p***
Age (y)	30.1	2.72	<0.001
Height (cm)	177.1	7.42	0.037
Weight (kg)	75.7	10.99	<0.001
BMI (${\mathrm{kg}/m}^{2}$)	24.1	3.15	<0.001

**Table 2:** Second phase participants’ anagraphic and anthropometric characteristics.

Descriptives			*Shapiro-Wilk*
	**Mean**	**SD**	***p***
Age (y)	24.0	4.35	<0.001
Height (m)	1.80	0.08	0.907
Weight (kg)	72.8	11.43	0.375
BMI (${\mathrm{kg}/m}^{2}$)	23.7	2.79	0.576

**Table 3:** Correlation matrix between height and Total Time ($RT_{TT}$) and Reaction Time Intertime ($RT_{Int}$). Note: *** p<0.001.

Correlation Matrix (Height)	${\mathrm{RT}}_{\mathrm{TT}}$	${\mathrm{RT}}_{\mathrm{Int}}$
*Mean ± SD*	49.30 ± 4.31	0.70 ± 0.10
*Spearman’s rho*	−0.482 ***	−0.475 ***
*Standardized β*	−0.54	−0.51
$R^{2}$	0.232	0.226

**Table 4:** Correlation matrix between weight and Total Time ($RT_{TT}$) and Reaction Time Intertime ($RT_{Int}$). Note: *** p<0.001.

Correlation Matrix (Weight)	${\mathrm{RT}}_{\mathrm{TT}}$	${\mathrm{RT}}_{\mathrm{Int}}$
*Mean ± SD*	49.30 ± 4.31	0.70 ± 0.10
*Spearman’s rho*	−0.222 ***	−0.217 ***
*Standardized β*	−0.24	−0.22
$R^{2}$	0.049	0.047

**Table 5:** Correlation matrix between BMI and Total Time ($RT_{TT}$) and Reaction Time Intertime ($RT_{Int}$).

Correlation Matrix (BMI)	${\mathrm{RT}}_{\mathrm{TT}}$	${\mathrm{RT}}_{\mathrm{Int}}$
*Mean ± SD*	49.30 ± 4.31	0.70 ± 0.10
*Spearman’s rho*	0.052	0.055
*Standardized β*	0.08	0.09
$R^{2}$	0.003	0.003

**Table 6:** Descriptive analysis results and paired sample *t*-test comparison between FP and AP. Note. ** *p* < 0.01.

Comparison		
	***FP***	***AP***
$RT_{TT}$ (s)	32.1 ± 3.26	30.0 ± 2.98 **
$RT_{Int}$ (s)	0.36 ± 0.057	0.30 ± 0.066 **

## Data Availability

The dataset generated and analyzed during the current study is not publicly available due to ethical and privacy constraints concerning participant confidentiality and compliance with applicable data protection regulations. However, anonymized data may be disclosed upon reasonable request to the corresponding author, subject to appropriate data sharing agreements and approval from the institutional ethics committee.

## Abbreviations

The following abbreviations are used in this manuscript:

FP	Fixed Panel
AP	Adjustable Panel
RT	Reaction Time
$RT_{TT}$	Reaction Time Total Time
$RT_{Int}$	Reaction Time Intertime
